# Improving lifespan automation for *Caenorhabditis elegans* by using image processing and a post-processing adaptive data filter

**DOI:** 10.1038/s41598-020-65619-4

**Published:** 2020-05-26

**Authors:** Joan Carles Puchalt, Antonio-José Sánchez-Salmerón, Eugenio Ivorra, Salvador Genovés Martínez, Roberto Martínez, Patricia Martorell Guerola

**Affiliations:** 1 Universitat Politècnica de Valéncia, Instituto de Automática e Informática Industrial, Valencia, Spain; 2 Cell Biology Laboratory/ADM Nutrition/Biopolis SL/Archer Daniels Midland, Paterna, Valencia, Spain

**Keywords:** Imaging and sensing, Optoelectronic devices and components, Optical sensors

## Abstract

Automated lifespan determination for *C. elegans* cultured in standard Petri dishes is challenging. Problems include occlusions of Petri dish edges, aggregation of worms, and accumulation of dirt (dust spots on lids) during assays, etc. This work presents a protocol for a lifespan assay, with two image-processing pipelines applied to different plate zones, and a new data post-processing method to solve the aforementioned problems. Specifically, certain steps in the culture protocol were taken to alleviate aggregation, occlusions, contamination, and condensation problems. This method is based on an active illumination system and facilitates automated image sequence analysis, does not need human threshold adjustments, and simplifies the techniques required to extract lifespan curves. In addition, two image-processing pipelines, applied to different plate zones, were employed for automated lifespan determination. The first image-processing pipeline was applied to a wall zone and used only pixel level information because worm size or shape features were unavailable in this zone. However, the second image-processing pipeline, applied to the plate centre, fused information at worm and pixel levels. Simple death event detection was used to automatically obtain lifespan curves from the image sequences that were captured once daily throughout the assay. Finally, a new post-processing method was applied to the extracted lifespan curves to filter errors. The experimental results showed that the errors in automated counting of live worms followed the Gaussian distribution with a mean of 2.91% and a standard deviation of ±12.73% per Petri plate. Post-processing reduced this error to 0.54 ± 8.18% per plate. The automated survival curve incurred an error of 4.62 ± 2.01%, while the post-process method reduced the lifespan curve error to approximately 2.24 ± 0.55%.

## Introduction

*Caenorhabditis elegans* (*C. elegans*) is a widely used animal model in biological research due to certain advantageous features for investigation^1,2^. *C. elegans* is small, which allows it to be stored, handled and fed very efficiently. It is transparent, which also makes it easy to observe.

Certain types of behaviour demonstrated by these nematodes may increase our understanding of other more complex animals. Consequently, assays are designed to analyse different issues such as the study of compound toxicity, neurodegenerative diseases, ageing alterations, etc. For ageing assays, the lifespan model is employed^3–9^, which counts live animals of the same age over their lifetime. These are separated into populations, each of which undergoes a differentiating condition that may alter the life expectancy of a given population. Worm movement indicates life whereas death is defined by a lack of motion after stimulation with a platinum wire. *C. elegans* lifespan is close to 3 weeks, and some strains may live a few weeks longer. Statistical assays, like lifespan, need circa 100 specimens per condition, which greatly increases the number of worms and hinders the technician’s task. Therefore, there is a need to automate such assays to save researchers’ time and to provide objectivity.

There are different methods to automate *C. elegans* inspection tasks. The most widespread method is to measure worm movement by acquiring images while fully monitoring standard Petri dishes^10–13^.

Lifespan automation is challenging because a host of problems can arise. The image processing software must be designed to avoid different causes of false-negatives (or undetected live worms) and false-positives (or wrongly detected live worms). False-negatives can be due to worm aggregation problems or to occluded plate zones (e.g. zones near plate walls, or non-transparent zones due to contamination or condensation problems). False-positives can be due to progeny, worm decomposition or dirt contamination problems.

Research groups have developed different culture protocols to avoid progeny and to alleviate worm aggregation, plate contamination and condensation problems. Active lighting techniques^14^ can also alleviate plate contamination and condensation issues. However, these protocols and methods have failed to fully eradicate these problems, and thus image-processing software must deal with all these complications.

Reviews^15–27^ show that many image-processing software tools have been developed to monitor different types of *C. elegans* behaviour. These tracker tools work differently to our proposed method. They extract certain predefined worm features (speed, body bends, etc.) from the image sequences captured by an image acquisition system. Consequently, they require complex algorithms and/or human assistance and supervision to achieve good results. By contrast, our method extracts lifespan curves by using simple techniques that involve no human threshold adjustments or supervision.

For lifespan assays, we found the two following automated tools in the literature: the Lifespan Machine^12^ and WorMotel^28^.

On the one hand, WorMotel uses a robot-arm system to transport specific multi-well plates from a buffer cassette into an inspection zone and returns these plates to the buffer cassette after capturing an image sequence. Several image sequences can be captured daily by the same acquisition system for different assays. WorMotel avoids the worm aggregation problem because each well contains only one worm. Death event detection is based on a simple movement detection algorithm with image-processing differentiation. This method does not require a tracking algorithm because there is only one worm per well. On the other hand, Lifespan Machine is based on many scanners located inside incubators, which can run only one assay per scanner. In these machines, standard Petri plates are used and each plate can contain several worms. These plates are not moved during the assay, which is run to capture image sequences. In this case, each worm’s movements can be tracked before death because worms hardly move at the end of their lives.

This paper presents a different image-processing software system, based on an intelligent illumination system that is able to work in both the aforementioned image acquisition scenarios. In this case, the worm tracking problem is solved by using mechanical fixtures and image alignment techniques to correct any placing inaccuracies between the image sequences captured at several time points. We consider our method to be flexible as it can work in many different acquisition scenarios and is easily adaptable to other assays (e.g., healthspan or memory assays). For this reason it has a high frame acquisition to analyse young worm’s tracks (e.g. healthspan) and can compare these tracks among days (lifespan).

The shared objective of all these tools is to base death event detection on the last movement detected for a tracked worm. However, false-positives can be detected due to the worm decomposition process, dirt contamination or image alignment errors. False-negatives may also be detected due to worm aggregation and occluded zones.

Lifespan curves are monotonically decreasing functions. Therefore, a lifespan-counting error can be detected when the current live-worm count is higher than a previous count. In this paper, a post-processing adaptive data filter is proposed to correct all the detected lifespan counting errors by taking into account error incidence probabilities to improve lifespan determination results. This technique has been evaluated for lifespan (the most complex assay due to its long experimental duration) by taking images with controlled lighting based on active vision, which alleviates some errors by improving image quality^14^.

The main goal of this work is to demonstrate that a simple method is feasible to obtain lifespan curves by using simple movement detection and filter algorithms when images are captured by an active illumination system. The results demonstrate that lifespan curves were automatically extracted using a specific lifespan assay protocol and two image-processing pipelines applied to different plate zones. Finally, our experiments demonstrated that the new adaptive data post-processing method reduced the initial alive count errors from approximately 4.62 ± 2.01% to 2.24 ± 0.55% per lifespan curve.

## Methods

### *C. elegans* strains and culture conditions

*C. elegans* strains N2, Bristol (wild-type) and CB1370, *daf-2 (e1370)* were obtained from the Caenorhabditis Genetics Center at the University of Minnesota. All strains were maintained at 20 °C on nematode growth medium (NGM) seeded with strain OP50 of *Escherichia coli* as a standard diet.

### A specific lifespan assay protocol

Lifespan assays were performed with wild-type strain N2 or *daf-2* (insulin receptor). The age- synchronised worms were obtained by hatching the eggs from gravid worms in NGM plates of 55 mm diameter, and incubating at 20 °C until reaching the young adult stage. FUdR (0.2 mM) was used to prevent reproduction which impacts animal lifespans^29^, and fungizone (1 *μg*/*mL*) was added to prevent fungal contamination. The plates with fungal contamination were censored, following standard methods^30^.

The following specific culture protocol items were established to alleviate worm aggregation, contamination, plate wall occlusions, and condensation problems:

*C. elegans* strains (N2 strain and *daf-2*) were used, which do not display aggregation behaviour^31,32^. In order to lower worm aggregation probability, only 10 to 15 worms were cultured in each Petri plate. In this scenario, the aggregation probability was very low, and decreased with each assay day because the number of live worms decreased.

Petri plates were closed with a lid and an anti-fungal agent (fungizone) was added to reduce contamination. The *E. coli* OP50 lawn was seeded in the middle of the plate as worms tend to stay on the lawn, thus avoiding occluded wall zones. On each assay day, a human operator removed a small set of plates from the incubator and placed each one or more inside one image acquisition systems to capture and save an image sequence per plate. An image sequence consisted of 30 images acquired at 1 fps. Therefore, the time that Petri plates were outside the incubator was quite short, thus, avoiding condensation problems. Room temperature was maintained close to 20 °*C* to prevent condensation, which is produced by temperature changes. If condensation was detected, it was manually eliminated by the human operator before the image acquisition process commenced.

### Lighting system method

Different lighting techniques can be applied to monitor worms cultured on standard Petri plates. These techniques are defined by location in relation to the lighting device, the inspected plate and the camera. A backlight configuration consists of placing a camera in front of the lighting system and the inspected plate in between. In this case, both Petri plates and media must be transparent. Backlight illumination obtains high-contrast images with dark *C. elegans* and a bright background.

Active Backlight illumination^14^ was used by the image acquisition system to alleviate contamination, plate wall occlusion and condensation problems. It controls grey levels in images by keeping the background and worm grey levels within the same range of values. As demonstrated in^14^, active Backlight illumination is more robust in the presence of contamination and condensation problems than standard backlight illumination systems. The compensated images show a higher Fisher index (0.8636 ± 0.1427) than the non-compensated images (0.2049 ± 0.0267). It is important to remark that the proposed method is based on this active illumination system, which reduces the variability of the captured images. This smart acquisition system facilities automated image sequence analysis, does not need human threshold adjustments and simplifies the techniques required to extract lifespan curves.

### Image acquisition method

**Figure 1:**
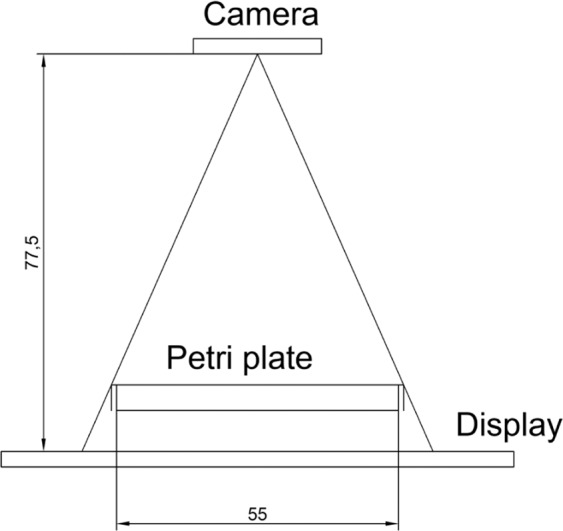
System physical configuration. Camera is above, backlight below with Petri dishes placed between them. Units are in mm.

The image acquisition method was replicated exactly from the Active Backlight illumination referred to in the previous point^14^, which consists of an RGB Raspberry Pi camera v1.3, a 7′′ Raspberry Pi display and a Raspberry Pi 3 as a processor. The element configuration (Fig. 1) places the camera above, display as illumination system below with the Petri dish placed between them. The camera sensor is OmniVision OV5647, which has a resolution of 2592 × 1944 pixels, a pixel size of 1.4 × 1.4 *μm*, a view field of 53.50° × 41.41° and the original lens with optical size 1/4′′ and 2.9 of focal ratio. The distance between camera and object (Petri plate) was sufficient to enable a complete picture of the Petri plate (about 77 mm), and the camera lens was focused at this distance. The 7′′ display has an 800 × 480 resolution at 60 fps, 24-bit RGB colour. Image sequences were taken to be processed to detect live worms, 30 seconds of 1944 × 1944@1 Hz, which means 30-image sequences. One image sequence per plate was taken daily and images were processed. Plates were placed in a vision system. Then the image sequence was taken and replaced with the next plate for inspection.

The acquisition system is open-hardware. The guidelines to build this system and the assembly description can be found in previous work^14^.

### Different image-processing pipelines applied to two plate zones

**Figure 2:**
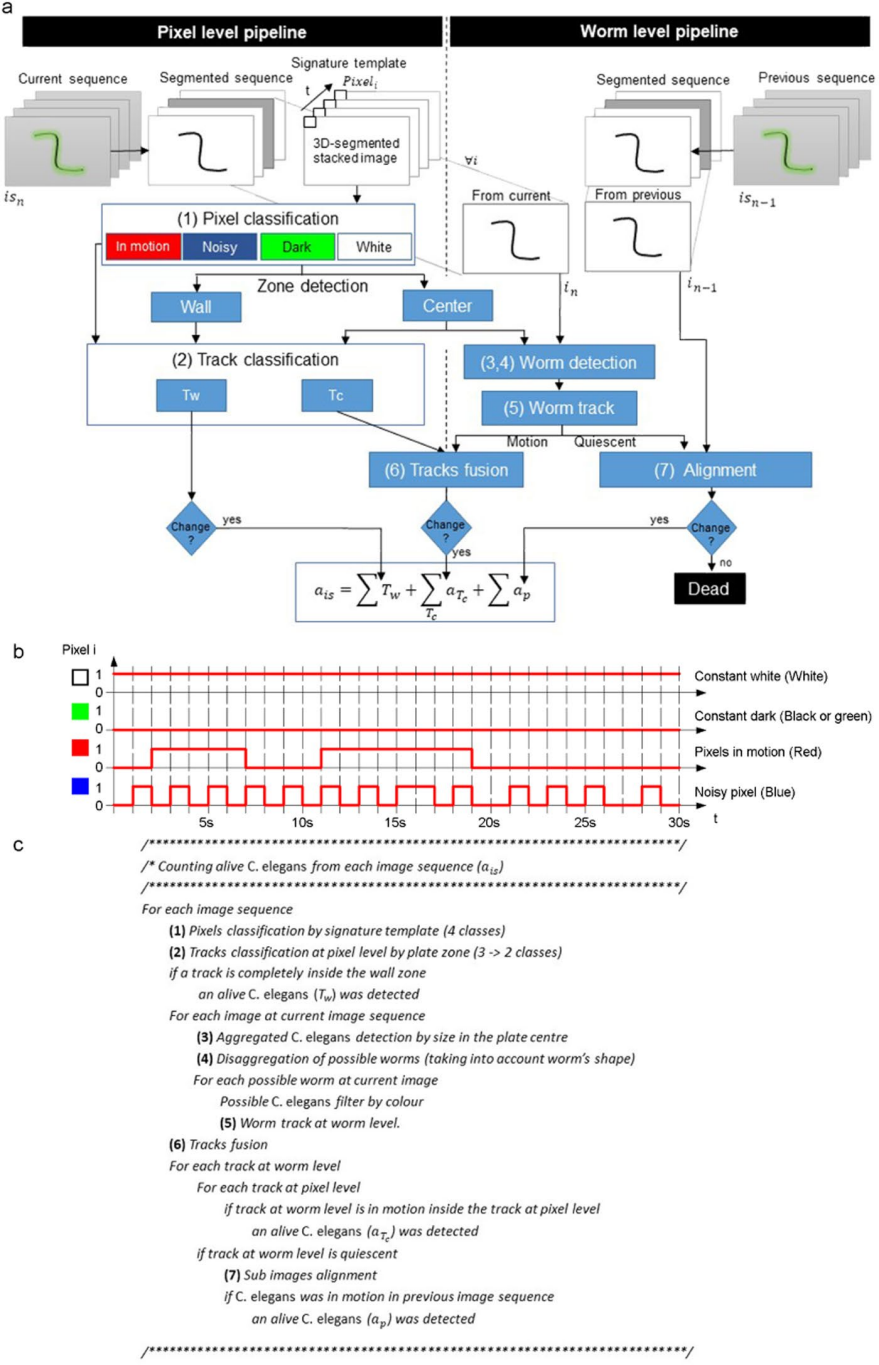
Image-processing pipelines. (**a**) Algorithm flowchart. (**b**) Pixel level. Signature template examples for the four possible cases. (**c**) Pseudo-code algorithm.

Death event detection (Fig. 2a,c) was defined when a worm did not move during a 24-hour period. This means that no movement was detected between the image sequence captured on one day and the image sequence captured the day before. Motion can be detected in an image sequence (composed of 30 continuous images, captured for 30 seconds) or between image sequences captured on different days. This simple criterion allows lifespan curves to be recalculated every day during the assay.

The proposed techniques were applied at two different levels (Fig. 2a). On the one hand, techniques at the pixel level used one-pixel features. At this level, the proposed method used temporal signatures (Fig. 2b). The temporal signature of one pixel consisted of the concatenation of all the segmented grey values of that particular pixel in the temporal sequence. On the other hand, techniques employed at the worm level used the features of a set of connected pixels belonging to a worm in one image (blob features).

Regarding worm detection complexity, plates presented two different zones due to their very distinct illumination conditions. While the plate centre presented a homogeneous illumination zone, the wall zone presented dark rings and many noisy pixels. In our case, the active illumination system created some well-illuminated white rings in the wall zone, where worms in motion were detected by simple techniques at the pixel level. Tracks at the pixel level were detected in the whole plate. However, throughout the 30-image sequence, the worms were tracked only in the central zone. Therefore, redundant information about the tracks at these two levels was found in the central zone. This allows tracks to be fused, in order to avoid inconsistencies and count how many tracks were moving at the worm level at the same time inside each track at the pixel level.

**Figure 3:**
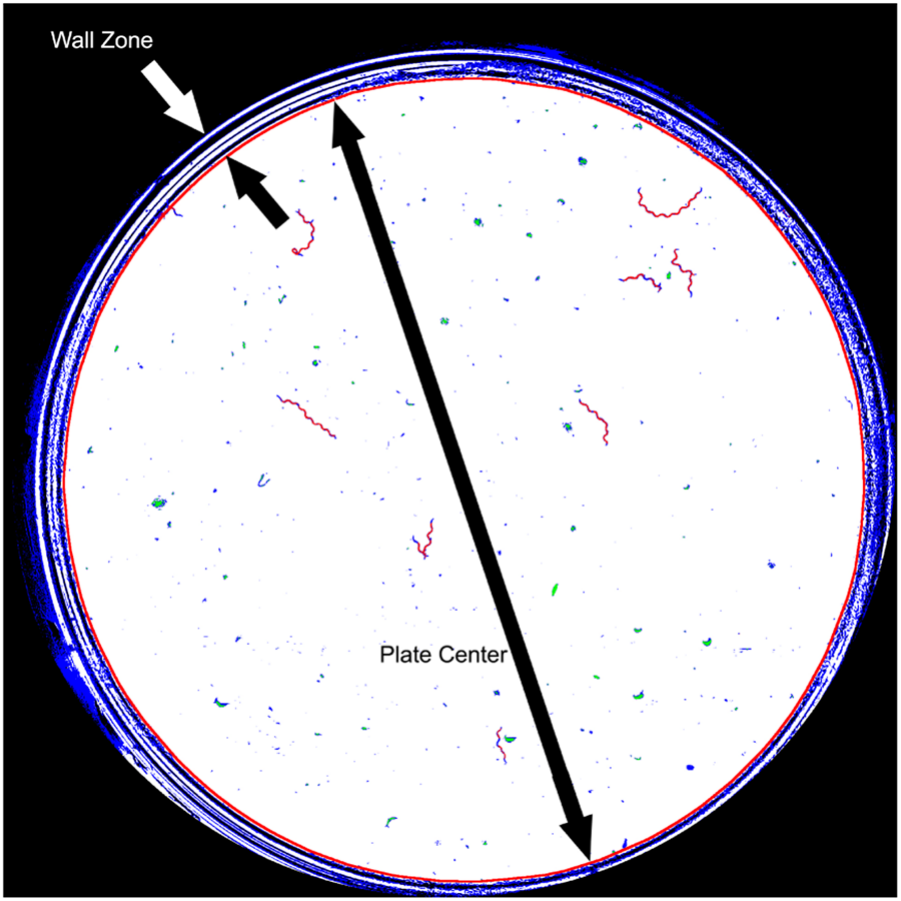
Classification at pixel level. The red circumference marks the edge between the two plate zones.

Given light refraction on walls, our captured images presented some dark and narrow rings (Fig. 3) in the wall zone. Therefore, only a motion analysis at the pixel level was possible in this zone. An image-processing pipeline based on a simple movement-detection algorithm at the pixel level was applied near the wall zone. However in the centre of the plate, a motion analysis at pixel level was fused with the motion analysis at worm level.

The first step consisted of classifying pixels by their signature templates (Fig. 2b). This step involved pixel segmentation per image by taking a fixed 33 grey intensity threshold and avoiding any manual threshold adjustment. This fixed threshold segmentation procedure was possible because the background pixels were controlled as being close to grey level 48 by an active lighting system^14^. Afterwards, the 30-segmented images were stacked in one 30-channel image. Each pixel in the stacked image was classified as a ‘constant dark’ pixel (black or green), a ‘constant white’ pixel (white), a ‘noisy pixel’ (blue) or a ‘pixel in motion’ (red), depending on the temporal signature (Fig. 2b). The temporal signature of a pixel was composed of its 30 stacked values. If all the values were black, this pixel was classified as ‘constant dark’. If all the values were white, it was classified as ‘constant white’. ‘Noisy pixels’ and ‘pixels in motion’ presented different patterns switching between black and white. Specifically, ‘noisy pixels’ presented a higher frequency of changes than pixels in motion.

Afterwards, the central plate zone was detected automatically by selecting the white blob with the maximum area. The contour of this blob was the edge between the plate centre and the wall zone. Constant dark pixels were black-coded in the wall zone and green-coded in the plate centre. The result of these steps is shown in Fig. 3.

**Figure 4:**
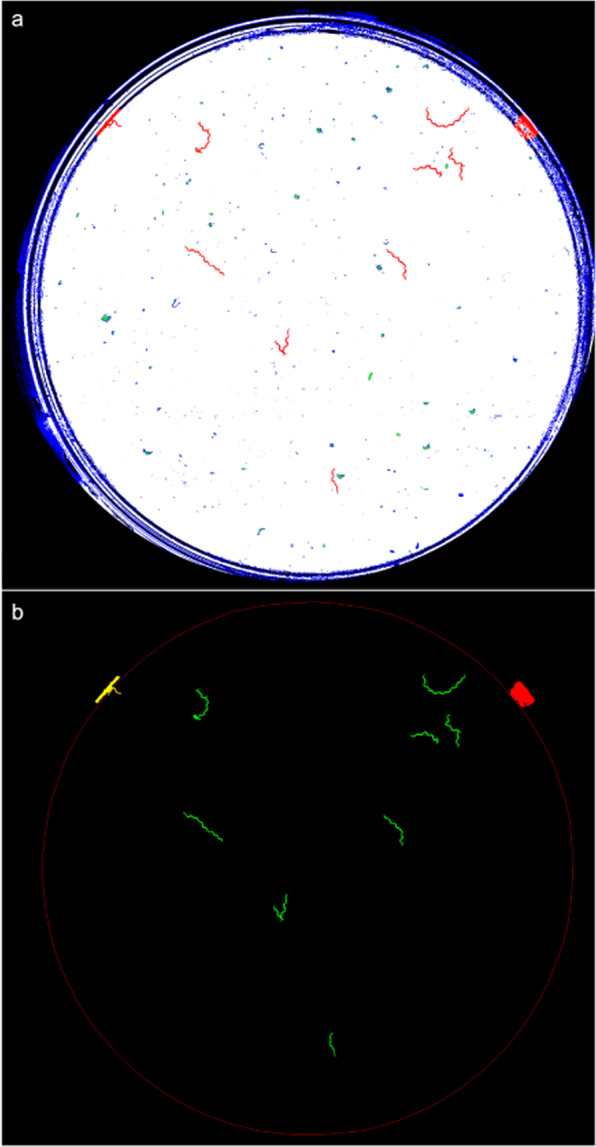
Detection of movement and classification. (**a**) Blobs representing detected tracks (marked in red). (**b**) The contours of the classified tracks at pixel level.

The second step consisted of classifying movements following plate zone criteria (Fig. 4). In this step, the red pixels in the wall zone were dilated to a radius of 40 pixels through the black and blue pixels. The purpose of this dilation was to connect the pixels in motion going through the black rings (cast by wall shadows and reflections) present in the wall zone. Next connected components labelling was applied by considering red, green and blue pixels to be equivalent colours in the plate centre and only red pixels in the wall zone. These steps resulted in tracks at the pixel level (Fig. 4a). They all had some pixels in motion, which means that at least one live worm was moving in each track. Finally, these tracks were classified into three classes (Fig. 4b) depending on whether they were completely inside the wall zone (depicted in red), completely in the plate centre (depicted in green) or in between (depicted in yellow). The tracks completely inside the wall zone were denoted *T*_*w*_ and those in between or completely in the plate centre were denoted *T*_*c*_.

The image-processing pipeline in the wall zone was completed in this step. In this case, each movement fully detected in the wall zone was counted as one live worm because motions were detected at the pixel level. In this zone, some false-negatives could occur due to occlusion in the black (darkened by wall shadows) and blue (noisy zone) rings. In the plate centre however, the pipeline was developed as far as the worm level for each image.

**Figure 5:**
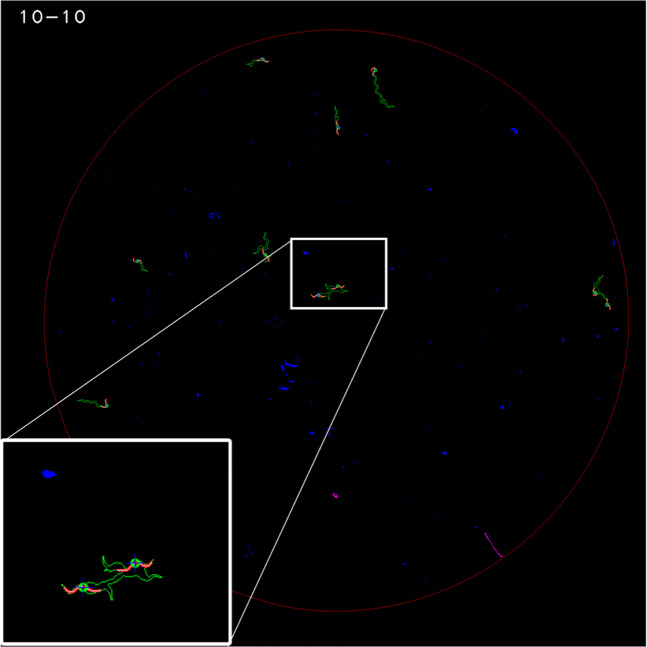
An example of a segmented image. The red, blue and magenta blobs represent the segmented results.

The third step started by performing connected components labelling (dark blobs) for each segmented image after taking into account only the plate centre (Fig. 5). These blobs (marked in red and magenta; discarded worms) represented possible aggregates formed by *C. elegans* and dirt contamination (marked in blue). They were filtered according to size by allowing sizes only from 20 pixels (the smallest detected worm area) to 240 pixels (the biggest detected area of two aggregated worms). In our case, some false-negatives could occur due to the aggregation of more than two worms during image sequencing.

**Figure 6:**
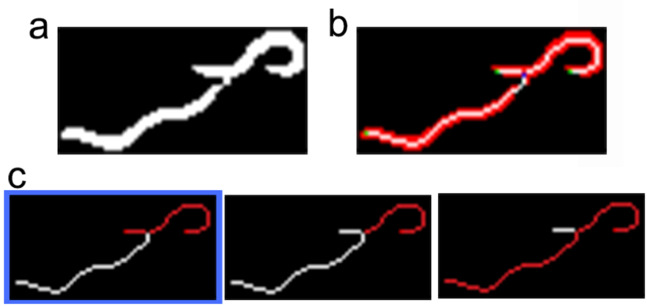
An example of two aggregated worms. (**a**) Segmented subimage. (**b**) Three skeleton edges (white), two end-points (green) and one cross (blue). (**c**) Searching space: three possible solutions (the *I*_*s*_ cost is lower in the first case, marked in blue).

The fourth step involved the disaggregation of two possible aggregated worms (Fig. 6a). This step started by extracting all the end-points (green points) and crosses (blue points) of each blob skeleton (white) (Fig. 6b). An optimisation algorithm was applied to extract all the possible disaggregated worms by considering shape features (length and width). A possible worm (*p*_*w*_) was considered a series of end-points and/or crosses connected by continuous skeleton edges. A set of possible worms (*s*) was a partition of continuous skeleton edges (possible worms (Fig. 6c)). The cost of a possible worm (*D*(*p*_*w*_)) was the Euclidean distance between the measured length and the width features of a possible worm with its theoretical values. The cost of a set (*I*_*s*_) (Eq. (1)) was the mean cost of all the costs of possible worms (*n*). After thoroughly exploring all the possible sets (*s*), the final selected set of disaggregated worms was the set with the minimum cost (Eq. (2)).

1 $${I}_{s}=\frac{\sum \,D({p}_{w})}{n}$$

2 $${\rm{\arg }}\,\min {I}_{s}$$

Then each possible disaggregated worm was filtered by colour features. Worm intensities were expected to be lower than the grey level 20. A detected worm was a blob that fulfilled the aforementioned shape and intensity features.

After detecting worms in the current image, the fifth step consisted of tracking worms along the 30-image sequence. Images were captured at 1 fps, which allowed worms to be more easily tracked because of their overlapping location between images. Then each worm-level track was classified as a motion or quiescent track.

The sixth step consisted of fusing the detected tracks at different levels to decide how many live worms were inside each pixel-level track. On the one hand, the pixel-level tracks (see Fig. 4b) were detected in the whole plate. On the other hand, the worm-level tracks were detected in the plate centre. This redundant information in the plate centre was used to avoid any inconsistency and to count how many worm-level tracks were in motion at the same time inside each pixel-level track. This count was the amount of live worms moving inside each pixel-level track (${a}_{{T}_{c}}$).

A live worm was a blob that accomplished the previous shape and intensity features, and its movement was detected in the last 24 h. It was classified as a live worm if motion was detected in either the current image sequence (${a}_{{T}_{c}}$) or between the current image sequence and the previous one (*a*_*p*_). The second option required comparing different image sequences. The seventh step consisted of image alignment techniques to correct any placing inaccuracies between the image sequences captured at different times.

At the end of their lives, worms do not change their location and hardly change their shape, but do move their heads. Therefore, it is easy to track worms at the end of their lives and thus detect death events, when only slight changes in shape can be expected. In this context, if a quiescent worm was detected along the current sequence of images, it was monitored to see if there were changes in the corresponding sub-image taken one day before. If there was no shape change, a high matching score would be expected between both sub images when allowing a small Euclidean transformation between them.

Finally, the live worm count was the sum of all the tracks completely inside the wall zone, all the live worms detected in each track completely or partially in the plate centre, and all the live worms for which motion was detected when comparing image sequences (Eq. (3)).

3 $${a}_{is}=\sum \,{T}_{w}+\sum _{{T}_{c}}\,{a}_{{T}_{c}}+\sum \,{a}_{p}$$

### Post-processing

Lifespan curves are monotonically decreasing functions. Therefore, a lifespan-counting error could be detected when a current live worm count (or in a current image sequence) was higher than a previous count. These errors can occur for two different reasons: (1) because the live worms detected in the current image sequence were aggregated or hidden in previous sequences (previous false-negatives) or (2) because some blobs, which erroneously appeared due to dirt contamination (dust spots on lids), met the live worm criterion in the current sequence (false-positives).

Herein, a new post-processing method is proposed in an attempt to optimally correct these errors. It is noteworthy that corrections were made only if a count error was detected in the automatically extracted lifespan curves. Corrective actions took into account error occurrence probabilities in order to act accordingly.

The initial number of live worms was known because the expert designed the experiment and placed the worms on the plates. Therefore, this initial value per plate can never exceed the lifespan. If it was exceeded for any plate, this plate count was limited to its initial number. Post-processing was applied individually to each Petri plate so that every plate count would be separately corrected from other counts.

**Figure 7:**
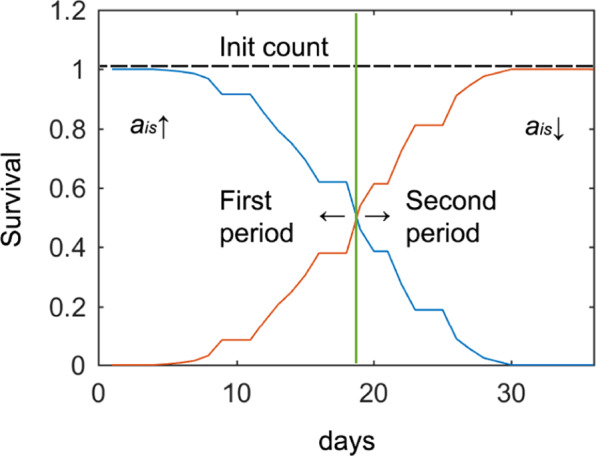
Two post-process stages or periods. Blue is the survival curve separated into the first (*a*_*is*_, value is corrected upwardly) and the second (*a*_*is*_, value is corrected downwardly) period by a dividing line on day 18. The red curve is the survival inverse (death curve).

In the first half of the lifespan cycle, more potential errors appeared due to hidden worms and aggregation (false-negatives) than to dirt (false-positives), and survival was high. In the second half of the lifespan cycle, this situation was inverted (dirt accumulated and survival dropped). Consequently, the post-process contemplated these two stages (Fig. 7).

On the plate edge, a shadow was cast by the plate wall, covering about 7% of the plate area in which worms are hidden. Although there was a low probability of the nematode being in that area, this error correction was made by post-processing. This meant that hidden worms were negative errors. Thus during the first cycle period, if a current image detected more worms than the previous day, it was interpreted as worms hiding in the shadow (or worm aggregation), and the previous day’s count was corrected by *a*_*is*_ being upwardly corrected (Fig. 7). It is unlikely that worms would remain hidden for more than one day, due to the small size of the shaded area and high mobility of young worms. During the second lifespan period, to reduce errors due to the aforementioned dirty environment, the post-processing strategy changed (positive errors had a higher probability than negative ones), and the currently detected worm count was less likely to be higher than the previous one. Therefore, the current count was the limit of the next day’s count (*a*_*is*_ is corrected downwardly). With this approach, the error standard deviation was reduced by half.

### Source code and lifespan experiment example

The source code is on github with an MIT open source license and the code repository is available (https://github.com/AntonioJoseSanchezSalmeron/lifespandownload). MATLAB, OpenCV and Java in Windows 10 were used. The code is evaluated with MATLAB R2018b and Java 1.8, and can be downloaded and run by launching MATLAB files (lifespan.m and postprocess.m). There is a lifespan experiment example that can be downloaded from https://active-vision.ai2.upv.es/wp-content/uploads/2020/01/Lifespan18.zipdownload and the processed results https://active-vision.ai2.upv.es/wp-content/uploads/2020/01/Lifespan18_Results.zipdownload.

### Validation method

To quantify errors, the lifespan experiment was performed in a standard laboratory without cleanroom facilities. In these experiments, the live worm count was done in duplicate: one automated count and another manual count. The automated count was done using the image-processing techniques described above, while the manual count was done using the captured processed images to check for any automated count errors. Thus the manual curve had to be taken as a reference and the error was measured. Apart from providing to be a good approach, post-processing showed the manual, automated and post-processing curves.

### Experiments and results

As previously mentioned, false-negatives and false-positives can occur due to occlusions, aggregation and dirt contamination problems. Some experiments were performed to estimate the probabilities of these errors and to assess the new data post-processing method. Essentially, the lifespan assay was needed for evaluations, whose methods are described above, and it was run four times in four different experiments, three to study variability and one to study robustness to large errors. Each experiment comprised 20 plates (55 mm), each containing 10 nematodes of the N2 strain (*n* = 200). With the lifespan experiments, automated errors and post-processing errors were analysed on the survival curve. For the experiment to study robustness, some errors were forced by displacing plates during data acquisition in order to assess post-processing robustness. First in the lifespan experiments, collision detection was done to estimate the aggregation probability for several cases (experiments with 10, 30, 60 and 90 worms per plate) to evaluate the most suitable worm count per plate.

### Aggregation probability estimation

**Figure 8:**
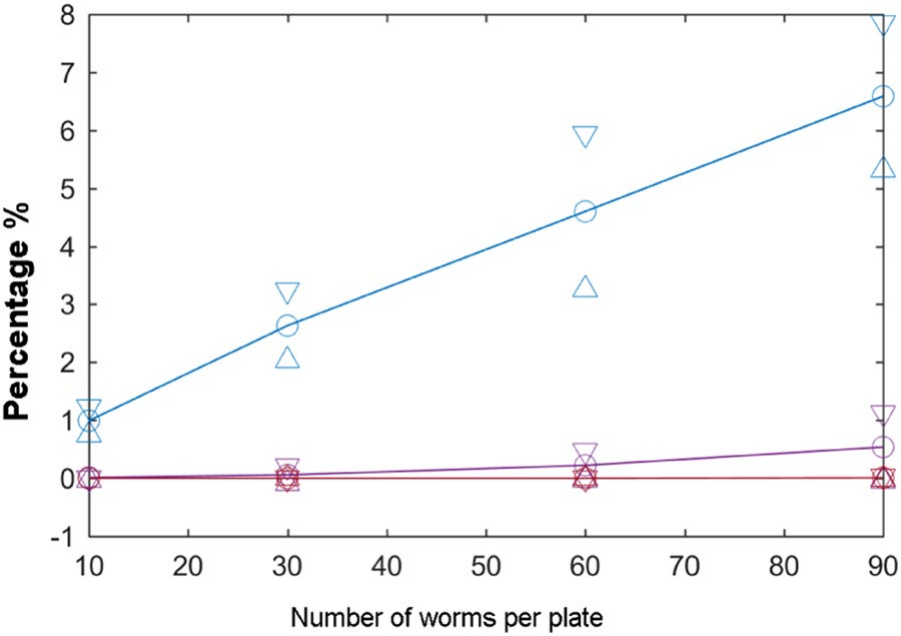
Aggregation probabilities. Influence of worm number per plate of 27.5-mm radius, where these curves are the probabilities of aggregation for two (blue line), three (purple line) and four (red line) worms.

In the lifespan experiments, worm population density is a widespread problem because clusters make it difficult to detect the exact number of nematodes. Both automated detection and human counts are difficult. The solution to this problem may be as simple as reducing the number of *C. elegans* individuals per plate. As determining an adequate number of worms per plate can be quite subjective, an attempt was made to estimate the aggregation probability depending on worm count. A lifespan assay with four conditions was performed for 30 seconds. Each condition had a worm count per plate that was a unique distinctive factor: 10 worms/plate (*n*_1_ = 360), 30 worms/plate (*n*_2_ = 360), 60 worms/plate (*n*_3_ = 360) and 90 worms/plate (*n*_4_ = 360). Collision events were calculated through the 30 images. As Fig. 8 shows, a 10-worm population caused a reasonable 1% aggregation of two worms, while 30, 60 or 90 individuals increased to 2.6 ± 0.6%, 4.6 ± 1.34% and 6.6 ± 1.26%, respectively. Thus a suitable choice would be 10 (or 15) worms on a 55 mm-diameter plate, which is 0.99 ± 0.24%. Hence we estimated that this percentage was low enough, and the experiments were conducted with this sample number per plate.

### False-positive and false-negative analyses per day

The four lifespan experiments were run in a normal laboratory where there were small lint and dust particles. The manual count of the captured images represents our ground truth. Nevertheless, wall shadows hid the worms even from human inspection, although worms were not hidden every day. Therefore, if a manual count was higher on one day than on the previous day, it would be reasonable to assume that worms were hidden in the wall area the day before. So human count regressive correction gave more realistic results, and two different curves (visible and real number of worms) were extracted from the human count, where the difference between these two counts gave the number of hidden worms, from which the probability of this event was calculated. Moreover, counting dirt errors allowed the likelihood of this event to be calculated.

**Figure 9:**
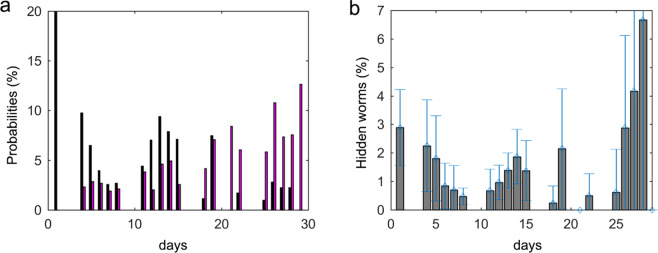
FN and FP probabilities per days. (**a**) Probabilities of false-negatives (black bars) and false-positives (magenta bars) per day. (**b**) Probability of worms being hidden on each day (they were located in the wall zone).

On the first lifespan assay days, the young L4 worms moved faster and covered longer distances than old worms on the last assay days. Both travelled distance and speed decreased with every passing day. This was why the probability of a live worm leaving an occlusion zone lowered with each assay day. However, dirt contamination accumulated with each assay day and, therefore, the probability of finding dirt contamination increased as the assay progressed. It was assumed that errors due to occlusions (false-negatives) were more probable than dirt contamination errors (false-positives) on the first assay days, and that errors due to dirt contamination (false-positives) were more probable than those due to occlusions (false-negatives) on the last assay days. The experiments confirmed that errors due to occlusions (false-negatives) were more probable than dirt contamination errors (false-positives) on the first assay days. Dirt contamination errors (false-positives) were more probable than those due to occlusions (false-negatives) on the last assay days. As shown in Fig. 9a, the probability per day of all the hidden events occurring was circa 20% on the first days and the false-positives rate was about 3% during the same period. During the second period, the positive errors increased to 12% and the negative errors dropped to 2%.

**Figure 10:**
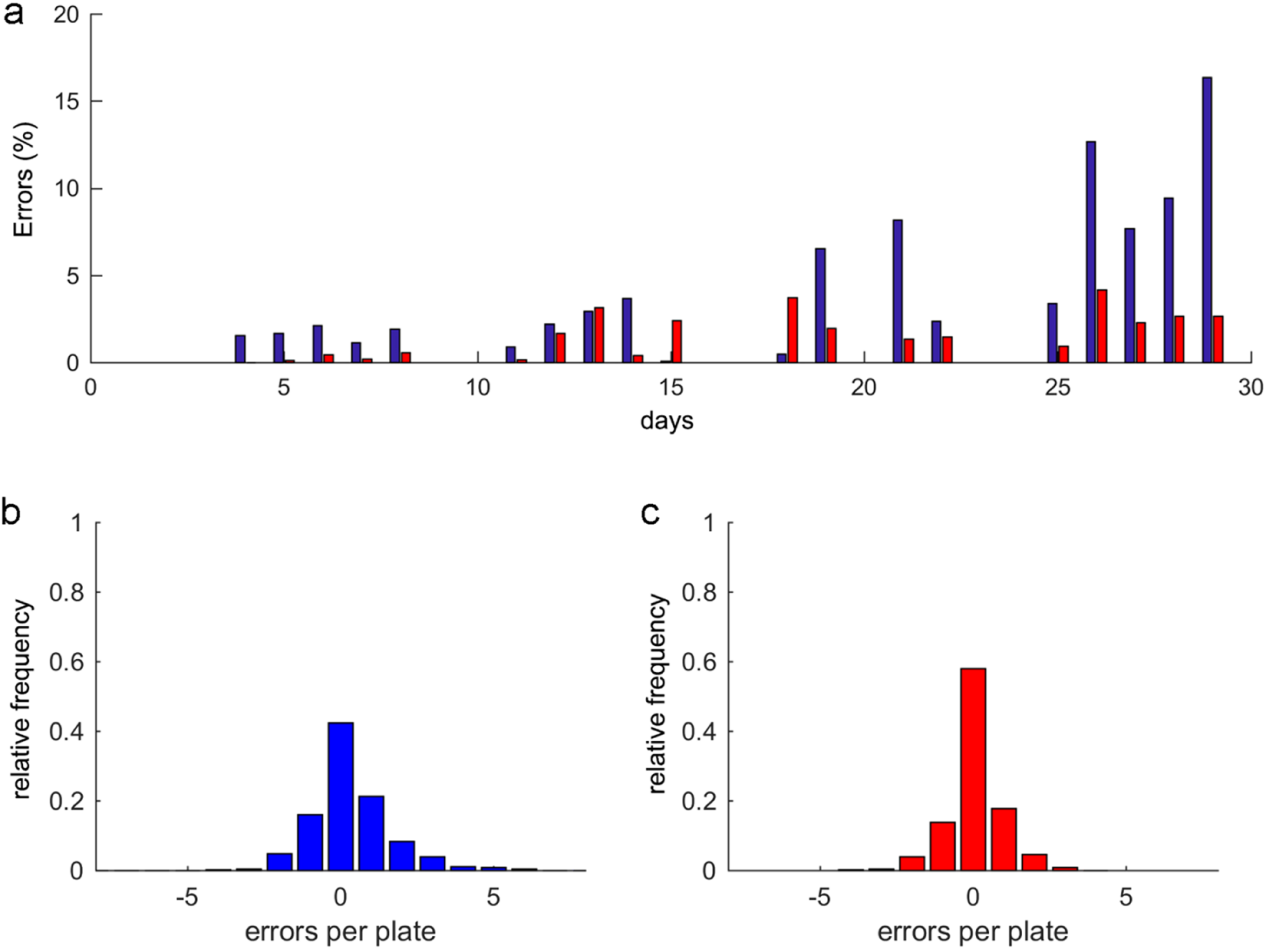
Error analysis. The error is *E* = Automated count – ground truth. Thus negative errors are non-detected worms (false-negative) and positive errors are detected contamination (false-positive). (**a**) is the absolute error percentage per day of the whole population, and the automated error (blue bars) is compared to the filtered result error by post-processing (red bars). (**b**) shows the automated error frequency distribution per plate for all three experiments with a positive or negative sign (in individuals), while (**c**) is the post-processing error frequency distribution.

Some false-negatives could be due to the aggregation of more than two worms during image sequencing. However, the probability of three worms being aggregated for 30 seconds (a complete image sequence) when 10 or 15 worms were cultured in a Petri plate was practically 0% (Fig. 8).

### No detection due to hidden zones

*E. coli* was sown in the plate centre so, as well-documented, worms will move near this feed zone. If we assumed random movement, 7% of worms would to be found on the plate’s edge (wall shadow) because this is the shadow area percentage. Nevertheless, as stated and Fig. 9b shows, the worm non-detection percentage by the edge zone was less than 3% almost every day. This better detection could be due to two phenomena, (1) worm speed, which facilities their movements to a large space within 30 seconds; (2) the previously stated animal nutritional needs. For the former, we were unable to differentiate between these two reasons for improved detection on the first days when we observed 3% of worms in the hidden zone. However after the mean life period had elapsed and when worms moved more slowly, we observed that the percentage of worms in the edge zone remained below 3%, which indicates that speed was also a reason for improved detection in this zone. On days 27 and 28, the percentage came closer to 7%, possibly because that worms moved quite slowly and *E. coli* may have run out in some plate zones.

### Comparing the automated and post-processing results

As stated previously, our ground truth was the corrected manual count lifespan curve, which was compared with the automated and post-processing curves. Figure 9 depicts how an optimum day near the mean lifespan can be used to change the post-processing filter strategy. A mean lifespan depends on conditions which may prolong or shorten it, and it usually takes place on day 14 with the N2 strain. A life versus death turning point occurs on this mean lifespan day (Fig. 7), meaning that there are fewer worms during the second period because some have died. For both these reasons, the period division day was selected as the mean lifespan of N2.

**Figure 11:**
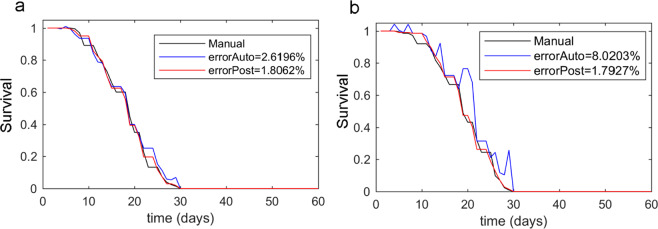
Lifespan curves. The lifespan curves measured manually (black line) and automatically (blue line), and the curve post-processing based on automated filtering (red line). (**a**) is a typical lifespan of the N2 strain. (**b**) is the N2 strain lifespan, but with some forced errors.

To assess this method, three lifespan experiments were conducted with the N2 strain, where each one composed 20 plates with circa 10 worms per plate. Experiments were performed following the methods described in this manuscript. The results are shown in Fig. 11a. These results indicated that an automated measurement had a typical error of 4.62 ± 2.01%. These values included errors due to contamination and dirt, which could vary according to room conditions. For all three experiments, post-processing always gave improved results by reducing the global error from 4.62 ± 2.01% to 2.24 ± 0.55%, and by the diminishing error spikes that spontaneously appeared at any time (Fig. 10c). Thus the post-process reduced not only the mean error, but also variability (Fig. 10a). This effect was observed more clearly in plates (Fig. 10b) where the automated error followed a Gaussian distribution of a mean of 0.32 individuals, a standard deviation of ±1.4 individuals and a non-error probability of 42%. When post-processing was applied, the mean error decreased to 0.06 individuals, the standard deviation to 0.9 individuals, but the non-error probability increased to 60% (Fig. 10c). This variability per plate according to standard deviation narrowed because high abnormalities of four or six errors were filtered. In order to check robustness, errors were produced on purpose by randomly forcing plate displacement during some captures, which gave false-positive errors (Fig. 11b). In this case, the automated error increased from 4.62% to 8.02%, but the post-processing algorithm was able to correct errors and reduced it to 1.8% error.

**Figure 12:**
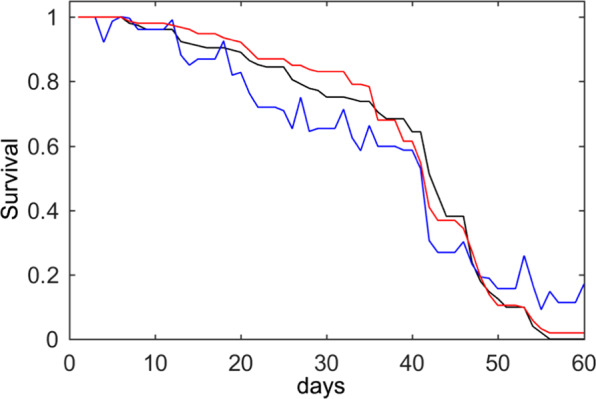
The *daf-2* survival curve. The black line is the manual count, the blue line is the automated count and the red line results from applying the post-processing method.

Another lifespan experiment was conducted following the same methods, but with *n* = 152 individuals and the *daf-2* strain with a longer lifespan, up to 60 days^33,34^. This number of days is considerably longer than that of the N2 lifespan, thus facilitating dirt accumulation. Therefore, it was of interest to study the *daf-2* strain lifespan applying this method. This longer life expectancy gave a distinct mean lifespan. Thus the daily life and death probabilities changed as regards both conditions and strains. As expected, the optimum selected day was also the mean lifespan (42), and the results revealed that this approach was correct. As Fig. 12 shows, errors higher than the N2 lifespan curves appeared, which was to be expected because contamination was higher. Nevertheless, the automated error was 8.54% and post-processing correction lowered this error to 3.43%.

## Discussion

The methods described herein were evaluated for the controlled illumination based on active vision, which maximized image quality and simplified image processing. These methods are also flexible as they can be applied to different acquisition scenarios, such as lifespan, healthspan, memory assays, etc. Furthermore, it is possible to conduct several experiments in parallel in the same device because plates can be replaced while data are being processed. Other methods, like Lifespan Machine, have the advantage of high throughput (but it cannot analyse other characteristics like healthspan) or WorMotel (which keeps worms isolated). The proposed method can be run in parallel with manual capture of image sequences, as in “Lifespan machine”, or even automated capture as in “WorMotel”.

A post-processing filter took into account the probability of errors occurring on different days by applying various error correction strategies to minimise final count errors. The optimal corrective action considered that all the errors on the first assay days were false-negatives and all the errors on the last days were false-positives. The results indicated that it was more advantageous to correct errors on the first assay days by taking them all to be false-negatives because their incidence probability was higher than for false-positives, and it would be more useful to correct errors on the last assay days by considering them all to be false-positives. This filter was applied independently of each plate.

The experimental results showed that the automated counting errors of live worms followed a Gaussian distribution, with a mean of 0.32 individuals (almost 0.0) and a standard deviation of ±1.4 individuals per Petri plate. Post-processing reduced this error to 0.06 ± 0.9% individuals per plate. The automated survival curve gave an error of 4.62 ± 2.01%, while the post-processing method reduced it to approximately 2.24 ± 0.55% of the curve error. Outliers were alleviated by this method which, hence, provided more robustness to plate displacements and dirty environments, and helped to ease typical occlusion problems due to aggregation, hiding, contamination or condensation. As the graphs reveal, the error average per plate came close to zero, hence: the larger the sample, the fewer errors on the lifespan curve. Thus by reducing errors with post-processing, the sample size is smaller for a given error, which implies shorter experimentation and analysis times, and cheaper costs in relation to plates, animals, storage, etc.

In order to reduce the negative errors caused by occlusions (wall shadows, aggregation, etc.) different tools and methods were developed and used. On the one hand, the motion analysis that worked at pixel level was applied to wall zones to detect live worms despite occluded dark rings. Theoretically, the probability of worms being inside this wall zone was 7%, but the results indicated that this probability was less than 3% because the feed was sown in the plate centre. On the other hand, the probability of three-worm aggregations came close to zero when using 10 to 15 worms on a 27.5 mm-radius plate. When a two-worm aggregation was detected (probability of 1.09%), an optimisation process was applied in an attempt to disaggregate them.

We studied two strains, N2 and *daf-2*, which were those assessed by this method. The results indicated that our post-processing method is a good lifespan determination method for any nematode strain, even for a long lifespan during which dirt and contamination progressively increase. It may also be suitable to set the mean lifespan as an optimum day to change the correction strategy.
